# Facile and reversible digestion and regeneration of zirconium-based metal-organic frameworks

**DOI:** 10.1038/s42004-019-0248-7

**Published:** 2020-01-09

**Authors:** Jun Chu, Fu-Sheng Ke, Yunxiao Wang, Xiangming Feng, Weihua Chen, Xinping Ai, Hanxi Yang, Yuliang Cao

**Affiliations:** 1 College of Chemistry and Molecular Science, Hubei Key Laboratory of Electrochemical Power Sources and Sauvage Center for Molecular Sciences, Wuhan University, 430072 Wuhan, China; 2 College of Chemistry and Molecular Engineering, Zhengzhou University, 450001 Zhengzhou, China

**Keywords:** Metal-organic frameworks, Porous materials

## Abstract

The digestion/regeneration of metal-organic frameworks (MOFs) has important applications for catalysis, drug delivery, environmental decontamination, and energy storage, among other applications. However, research in this direction is limited and very challenging. Here, we develop a facile method to digest and regenerate a series of zirconium-based metal-organic frameworks (Zr-MOFs) by bicarbonate or carbonate salts. As an example, UiO-66 demonstrates well the mechanism of reversible digestion/regeneration processes. By analyzing the digested zirconium species via X-ray diffraction, Fourier transform infrared spectroscopy and Raman scattering spectroscopy, a digestion mechanism based on the formation of dissoluble complexes [Zr_2_(OH)_2_(CO_3_)_4_]^2−^ is proposed. Impressively, ultrafine Pd nanoparticles can be extracted from Pd@PCN-224 via this strategy. This work, thus, may provide new insight for the development of renewable MOFs and their practical applications.

## Abstract (web summary)

The ability to digest and regenerate metal-organic frameworks is valuable for their application in catalysis and drug delivery, but challenging to achieve. Here, the authors develop a facile method to digest and regenerate a series of zirconium-based metal-organic frameworks using bicarbonate or carbonate salts.

## Introduction

Metal-organic frameworks (MOFs) are emerging porous materials that have shown promise in cancer therapy^1,2^, catalysis^3–5^, chemical sensing^6,7^, and energy-storage applications^8–11^, including as electrodes,^12–20^ separators^21,22^, and electrolytes^23^, due to their high surface area^24^, abundant ordered pores^25^, and tailored pore environment^26^. Zirconium-based MOFs (Zr-MOFs) are promising framework materials for practical applications because of their higher thermal and chemical stability compared with other MOFs^27,28^. However, the durable skeletons hinder their future development as matrices for nanoparticles^29,30^ or quantum dots, reusable ligands, drug delivery systems^31^, and the component analysis of multivariate Zr-MOFs, where the framework should be completely removed. Although Zr-MOF digestion has been studied in aqueous solution with strong acids or corrosive or oxidizing reagents (i.e., HCl, HNO_3_, HF, and CeF)^32–35^, they are difficult to thoroughly dissolve even under such harsh conditions, which restricts their practical applications. Meanwhile, the regeneration of MOFs may offer access to cost-efficient and eco-friendly applications, but only few MOFs (such as MOF-5 and HKUST-1) have been investigated^36–38^. Furthermore, the structures of these regenerated MOFs are unstable in moisture. Therefore, it is greatly needed to explore facile and efficient methods to digest and regenerate rigid MOFs, such as Zr-MOFs.

Here, we provide a facile, cost-efficient, and environmentally friendly approach for reversible digestion/regeneration of Zr-MOFs by use of bicarbonate or carbonate solutions. UiO-66, a classical Zr-MOF consisting of zirconium-oxo cluster and 1,4-benzenedicarboxylic acid (BDC), is selected to complete the digestion–regeneration circle. On the other hand, ligand recycling and Pd nanocatalyst synthesis can be achieved from Pd@PCN-224 (palladium nanoparticles are embedded in PCN-224, which is a Zr-MOF) based on this digestion approach. In addition, a digestion mechanism actuated by entropy change is discussed based on the formation of dissoluble complexes [Zr_2_(OH)_2_(CO_3_)_4_]^2−^ for Zr-MOF digestion process.

## Results and discussion

### Digestion and regeneration of UiO-66

**Fig. 1:**
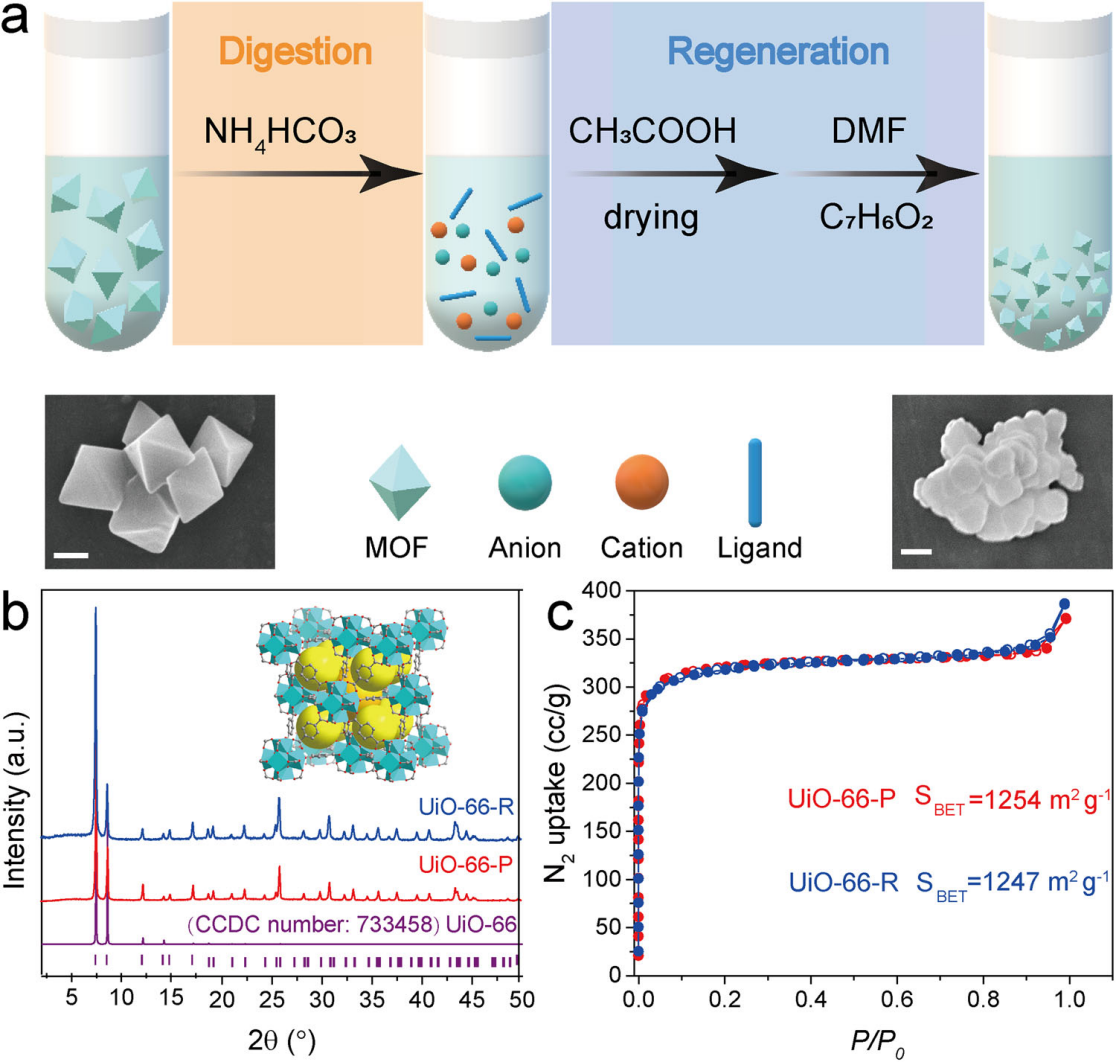
Conceptual and experimental diagram of digestion and regeneration of UiO-66. **a** Schematic illustration for digestion/regeneration process of Zr-MOFs with SEM images of the UiO-66-P (left) and UiO-66-R (right) (scale bar: 200 nm). **b** XRD patterns of the reported UiO-66 (purple, CCDC number: 733458), UiO-66-P (red), and UiO-66-R (blue, inset: structure of UiO-66). **c** Isotherms of N_2_ adsorption–desorption at 77 K (red: UiO-66-P; blue: UiO-66-R).

A typical digestion–regeneration process of Zr-MOFs is illustrated in Fig. 1a. When the pristine UiO-66 (denoted as UiO-66-P in following paragraphs) particles are immersed in ammonium bicarbonate aqueous solution at room temperature, a colorless transparent solution is observed, suggesting a successful digestion process. To realize the regeneration of the dissolved UiO-66-P, the pellucid solution is treated with acetate acid and then evaporated. The residual white powders could transform into UiO-66 crystals with the help of *N*,*N*-dimethylformamide (DMF) and benzoic acid, which follows the same synthesis conditions as those of UiO-66-P (the detailed experiment processes are described in the “Methods” section and Supplementary Methods). The regenerated UiO-66 (denoted as UiO-66-R) shows powder X-ray diffraction (XRD) peaks of UiO-66-P, all of which can be indexed to UiO-66 diffraction pattern^39^ in the Cambridge Crystallographic Data Center (CCDC) (Fig. 1b). Moreover, the isotherms of N_2_ adsorption–desorption at 77 K for UiO-66-P and UiO-66-R were acquired (Fig. 1c). UiO-66-R shows specific surface area of 1247 m^2^ g^−1^ and total pore volume of 0.59 cm^3^ g^−1^, respectively, very close to those of UiO-66-P (1254 m^2^ g^−1^ and 0.57 cm^3^ g^−1^, respectively). Meanwhile, the pore size distribution reveals that the UiO-66-P and UiO-66-R samples have the same micropore size of ~1 nm (see Supplementary Figs. 1 and 2). They also have identical chemical bonds according to the Fourier transform-infrared spectroscopy (FTIR) spectra (see Supplementary Fig. 3). Thus, it is anticipated that the UiO-66-R has the same properties with UiO-66-P, implying that a complete digestion/regeneration process of Zr-MOF is successfully realized. Besides, scanning electron microscopy (SEM) images in Fig. 1a show the agglomerated particle of UiO-66-R with the individual particle size of around 180 nm, which is smaller than UiO-66-P (around 300 nm). Moreover, it can be noted that the morphology of UiO-66-R is not perfect octahedral crystals as UiO-66-P, most likely due to the existence of water in reactant during regeneration reaction (see Supplementary Fig. 4)^40^. In fact, regenerated Zr-MOF inherits the original surface area and structure, it may show comparable performance in practical application. Furthermore, PCN-224 with valuable porphyrin-based ligand has been digested and regenerated (the XRD and SEM images are shown in Supplementary Fig. 5), which indicates the feasibility of this digestion/regeneration method for general Zr-MOFs.

### Factors influencing digestion

**Fig. 2:**
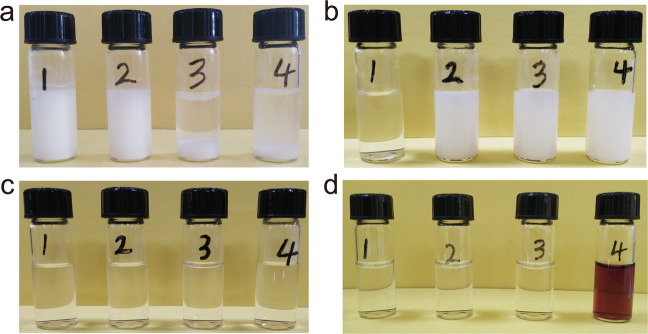
Photographs of UiO-66-P digestion. **a** UiO-66-P immersed in HCl (1), CH_3_COOH (2), NH_3_•H_2_O (3), and NaOH (4) aqueous solution. **b** UiO-66-P immersed in NH_4_HCO_3_ (1), NH_4_Cl (2), NH_4_NO_3_ (3), and (NH_4_)_3_PO_4_ (4) aqueous solution. **c** UiO-66-P immersed in NH_4_HCO_3_ (1), LiHCO_3_ (2), NaHCO_3_ (3), and KHCO_3_ (4). **d** UiO-66-COOH (1), UiO-66-NDC (2), UiO-66-Br (3), and PCN-224 (4) immersed in NH_4_HCO_3_ aqueous solution.

To understand the mechanism of Zr-MOF digestion, controlled experiments have been performed. First, the same mass of UiO-66-P was dispersed in HCl, CH_3_COOH, NH_3_•H_2_O, and NaOH solutions with 1 M concentration (Fig. 2a), respectively. In consistent with previous reports^31,41^, UiO-66-P can maintain its structure in neutral or acidic aqueous solution while being destroyed in alkaline solution (see Supplementary Fig. 6). But no matter what solution is tried, the degraded samples act as precipitates, illustrating that the pH value is not the reason for MOF digestion (further experiment is shown in Supplementary Fig. 8). Second, in order to reveal the effect of anions on the digestion of UiO-66-P, we replaced OH^−^ by HCO_3_^−^, Cl^−^, NO_3_^−^, and PO_4_^3−^ of ammonium salts, as displayed in Fig. 2b. Amazingly, UiO-66-P can only be digested in NH_4_HCO_3_ solution, implying that the HCO_3_^−^ lays a critical role in UiO-66-P digestion. Furthermore, to verify the function of HCO_3_^−^, three more bicarbonate aqueous solutions, that is, LiHCO_3_, NaHCO_3_, and KHCO_3_, were investigated (Fig. 2c). The results demonstrate that UiO-66-P can be completely dissolved in these bicarbonate aqueous solutions regardless of different cations. It further confirms that HCO_3_^−^ is the vital factor for the digestion process. In order to investigate the universality of the bicarbonate digestion method, Zr-MOFs with various organic ligands (their structures are shown in Supplementary Fig. 9), such as 1,2,4-benzenetricarboxylic acid (BDC-COOH), 1,4-naphthalene dicarboxylic acid (NDC), 2-bromobenzene-1,4-dicarboxylic acid (BDC-Br), and meso-*tetra*(4-carboxyphenyl)porphine (TCPP) (the corresponding MOFs are denoted by UiO-66-COOH, UiO-66-NDC, UiO-66-Br, and PCN-224) are employed to conduct the digestion experiment. All these Zr-MOFs can be degraded to form clear solution (Fig. 2d), which implies that this digestion process is determined by decomposition of zirconium-oxo clusters, but independent of the ligands. Thus, this digestion method is versatile for different Zr-MOFs, indicating a promising application of this digestion method in catalysis, drug delivery, and utilizing as matrices of nanoparticles.

### Zirconium species in digested solution

**Fig. 3:**
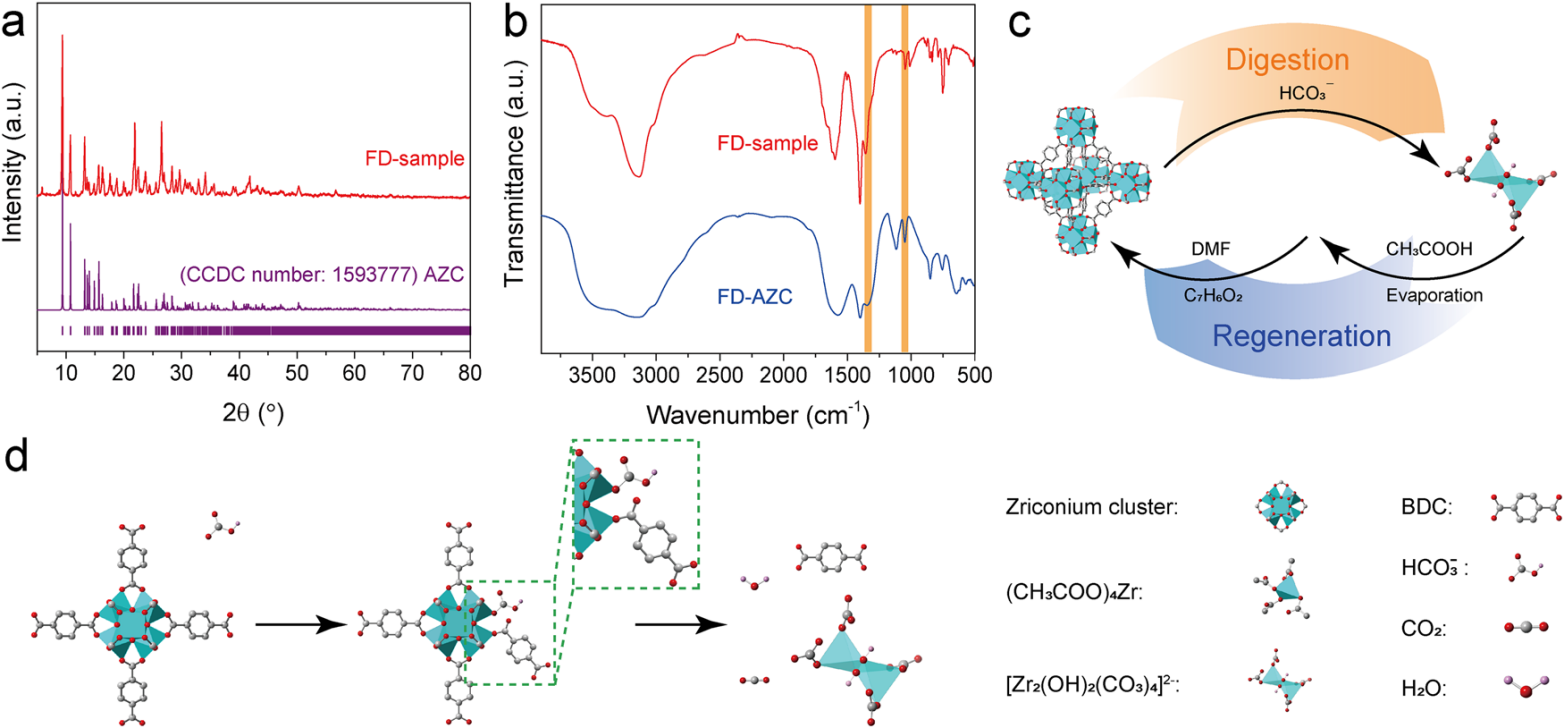
Characterization of FD sample and diagram of Zr-MOF digestion. **a** XRD patterns of FD sample (red) and reported AZC (purple, CCDC number: 1593777). **b** FTIR spectra of FD sample (red) and FD-AZC (blue). **c** Schematic illustration for the digestion/regeneration process of Zr-MOFs. **d** Schematic illustration of a competition–digestion mechanism for UiO-66 via bicarbonate-mediated approach.

In order to further understand the digestion mechanism of Zr-MOFs in bicarbonate aqueous solution, the digested zirconium species in the solution were systematically investigated. A digesting powder sample (denoted as FD sample) was gained by freeze drying (FD) the digested solution to prevent it from decomposing. Surprisingly, the dominated XRD peaks of FD sample in Fig. 3a are well matched with the reported diffraction pattern of ammonium zirconium carbonate (AZC) that is soluble in water, but unstable under high temperature^42^. This suggests that the carboxylic acid ligands and zirconium-oxo clusters transform into carboxylate ligands and AZC during the digestion process, respectively. Therefore, the AZC may be a possible digestion product of zirconium-oxo clusters. On the other hand, FTIR spectra were obtained to identify the chemical bond characteristics of the FD sample and FD-AZC powder (denoted as FD-AZC) (Fig. 3b). It is obvious that the FTIR feature of the FD sample is in good agreement with that of the FD-AZC, supporting the results of XRD (Fig. 3a). In addition, the bands (highlighted area in Fig. 3b) centered at 1356 and 1046 cm^−1^ reflect the symmetric stretch vibration of O–C–O and the stretch vibration of C–O of CO_3_^2−^ in AZC according to previous reports^43^. These FTIR evidences confirm the existence of AZC. Furthermore, the Raman spectra of FD sample and FD-AZC powders are shown in Supplementary Fig. 10. Obviously, the peak at 1052 cm^−1^ is assigned to the anion of AZC contained in CO_3_^2−^ and Zr species^44^. In addition, the Raman scattering signals of FD-AZC can all be found in the spectra of FD sample, which undoubtedly indicates the presence of AZC in solution.

Furthermore, proton nuclear magnetic resonance (^1^H NMR) was utilized for the sake of clarifying the change in ligand (see Supplementary Fig. 11). The single peak located at around δ = 7.7 indicated the presence of terephthalate in D_2_O. It revealed that BDC is released from UiO-66 with the existence of NH_4_HCO_3_, while it retained in the framework without the presence of bicarbonate. Therefore, UiO-66 is doubtlessly digested in NH_4_HCO_3_ aqueous solution.

### Mechanism of Zr-MOF digestion

Based on the above discussion, a MOF digestion–regeneration cycle was promoted with a view to zirconium species evolution (Fig. 3c). First, zirconium-oxo clusters, [Zr_6_O_4_(OH)_4_], exist in UiO-66 as metal sites. After adding NH_4_HCO_3_ aqueous solution, the soluble AZC is formed instead of zirconium-based clusters. Then, through the treatment of acetic acid and evaporation processes, UiO-66-R is emerged in DMF with the benzoic acid as a modulator via solvothermal method. Figure 3d shows a hypothetical competition–digestion mechanism of UiO-66. Like the deterioration mechanism of H_2_O or OH^−^^41^, zirconium-oxo cluster is subject to the attack of HCO_3_^−^, which takes place of the ligands coordinating with zirconium atoms. The ligands with carboxyl groups transfer into the corresponding salts; meanwhile, small molecules such as H_2_O and CO_2_ are generated. Because the structures of AZC are various and sensitive to the environment^44^, we denoted herein the anions of AZC by [Zr_2_(OH)_2_(CO_3_)_4_]^2−^ based on the above results^42^. Thus, the chemical reaction for UiO-66 digestion could be written as

1 $${\mathrm{Zr}}_6{\mathrm{O}}_4\left( {{\mathrm{OH}}} \right)_4\left( {{\mathrm{BDC}}} \right)_6 + \, 18\,{\mathrm{HCO}}_3^ - \to 3\left[ {{\mathrm{Zr}}_2\left( {{\mathrm{OH}}} \right)_2\left( {{\mathrm{CO}}_3} \right)_4} \right]^{2 - }\\ + \, 8\,{\mathrm{H}}_2{\mathrm{O}} + 6\,{\mathrm{CO}}_2 + 6\,{\mathrm{BDC}}^{2-}.$$

In addition, carbonate and citrate solutions can also digest UiO-66-P (see Supplementary Fig. 12a, the possible mechanism is shown in Supplementary Note 1), suggesting that forming a soluble complex with zirconium-oxo ions is a vital factor for the digestion of the UiO-66. Interestingly, we noted that the rate for digestion via carbonate and bicarbonate is much faster than that of citrate (see Supplementary Fig. 12b, c). The time for UiO-66-P completely digested in NH_4_HCO_3_ solution to form the transparent solution is within 20 min (see Supplementary Fig. 12b), whereas in ammonium citrate solution it is up to 3 days (see Supplementary Fig. 12c). This phenomenon can be explained by Eyring equation

2 $$\begin{array}{l}k = \left( {k_{\mathrm{B}}T/h} \right)\left[ {\exp \,\left( { - \Delta G^\# /RT} \right)} \right] = \left( {k_{\mathrm{B}}T/h} \right)\left[ {\exp \left( {\Delta S^\# /R} \right)} \right]\\ \hskip -10pc\left[ {\exp \left( { - \Delta H^\# /RT} \right)} \right],\end{array}$$

where *k* is the kinetic constant, *k*_B_ is the Boltzmann constant, *R* is the gas constant, *T* is the absolute temperature, *h* is the Planck constant, Δ*G*^≠^ is the activation free energy, Δ*H*^≠^ is the activation enthalpy, and Δ*S*^≠^ the activation entropy. It indicates that the large kinetic constant (*k*) owes to the high activation entropy (Δ*S*^≠^). Thus, the digestion process performed in bicarbonate and carbonate aqueous solution produces much smaller molecules, especially the gas molecules (e.g., CO_2_, Eq. 1, see Supplementary Eq. S1) than that in citrate aqueous solution (see Supplementary Eq. S4), resulting in a larger Δ*S*^≠^. Thus, the reaction rate in bicarbonate and carbonate solution is faster. Therefore, a competition–digestion mechanism actuated by entropy change could be suggested: Zr-MOF digestion occurred via the competing reactions between guest molecules and ligand molecules to form soluble species with metal-oxo clusters and low-molecular-weight products. For Zr-MOF digestion, three issues ought to be noticed: (1) the coordination ability of guest molecules should be stronger than ligands; (2) the digestion products are soluble in solvent; (3) generation of small-molecule products, particularly gases, can be beneficial to the digestion process due to larger entropy change.

Furthermore, for the regeneration process, a probable mechanism is proposed according to the formation of UiO-66^45–47^. After acid treatment and removal of CH_3_COONH_4_, NH_4_HCO_3_, and H_2_O by heat, the BDC and Zr(CH_3_COO)_4_ are left. Then, Zr-based compounds are formed by coordinating with CH_3_COO–, benzoic acid, and H_2_O in solvothermal procedure. Subsequently, UiO-66-R is generated via the ligand exchange between BDC and the ligands of Zr complex compound. The possible chemical reactions during regeneration of UiO-66 are suggested in Supplementary Note 2.

### Pd nanoparticles and ligand extraction

**Fig. 4:**
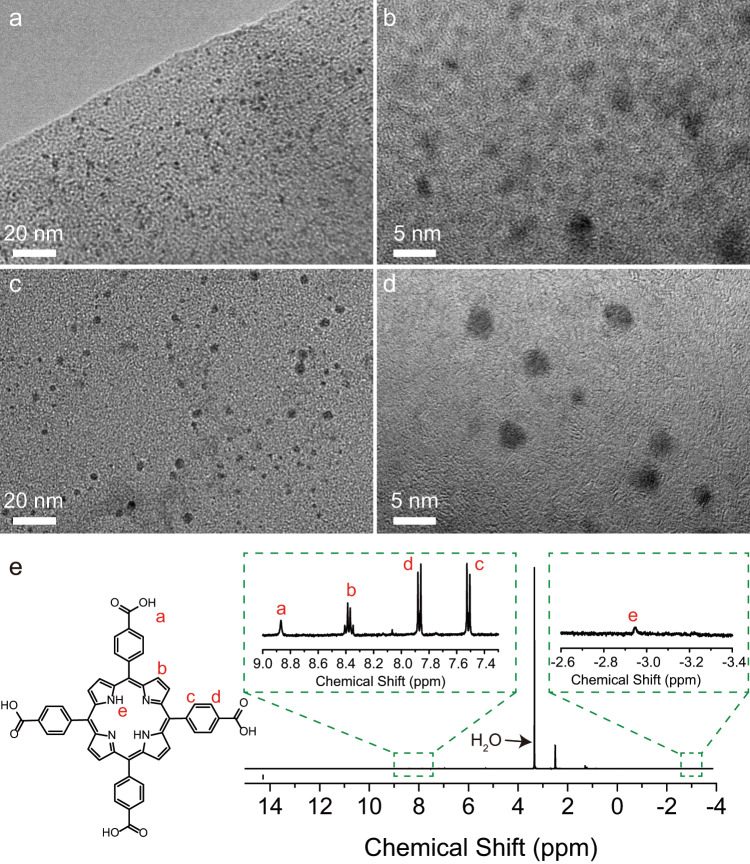
Pd nanoparticles and ligand extraction from Pd@PCN-224. **a**, **b** TEM images of Pd@PCN-224. **c**, **d** TEM images of Pd nanoparticles extracted from the digested Pd@PCN-224 solution. **e** ^1^H NMR spectra of recycled TCPP.

PCN-224, with Zr-based cluster and TCPP ligand, is a promising template and catalyst because of its applicable pore size and functional porphyrin-based ligand^48–50^. Hence, to further verify the application feasibility of the digestion process, Pd@PCN-224 (see Supplementary Fig. 7) was utilized for metal nanoparticle retrieval and ligand recycle. Figure 4a, b shows clearly well-dispersed Pd nanoparticles (2–3 nm) in Pd@PCN-224. After treating with 0.1 M NH_4_HCO_3_ solution, Pd@PCN-224 is digested and the nanoparticles are successfully exposed from the dissolved frameworks (Fig. 4c, d). It can be obviously found that the Pd nanoparticles extracted from PCN-224 retain their pristine size and morphology, without any aggregation (Fig. 4c, d). In addition, the TCPP ligand recycle experiment was also performed on PCN-224. The TCPP ligand obtained by PCN-224 digestion and acid treatment was verified by ^1^H NMR spectra (Fig. 4e). It indicates that this digestion method is feasible and has great potential applications in the synthesis of single-dispersed metal catalysts, catalyst recycle, and regeneration of expensive MOFs.

In conclusion, a facile, cost-efficient, environmentally friendly method for Zr-MOF digestion/regeneration has been developed by utilizing bicarbonate/carbonate solution. As a result, the digested products are all soluble in water, and the regenerated MOF samples have the same structure, specific surface area, and pore structure with their parent MOFs. Well-dispersed Pd nanoparticles were obtained by the digestion process of Pd@PCN-224 to verify the feasibility of the method. To our knowledge, it is the first case of the complete digestion/regeneration of Zr-MOFs. Besides, by analyzing the dynamics of digestion process in bicarbonate and citrate solution, a competition–digestion mechanism actuated by entropy change has been promoted. This facile digestion procedure provides a novel insight for not only ligand recycle, component analysis, nanoparticle synthesis, and drug release but also chemical sensors, extraction–separation, and synthesis materials with unique nanostructure. Although the further optimization and exploration of regeneration process are needed, this novel digestion/regeneration method sheds light on a general MOF digestion and regeneration process and provides access to future applications of MOFs.

## Methods

### Synthesis of UiO-66-X (X = P, COOH, Br, and NDC)

The mixture of BDC (or BDC-COOH, BDC-Br, and NDC), ZrOCl_2_•8H_2_O, benzoic acid, and DMF (mole ratio = 2:1:30:91) were sealed in an autoclave. After heating in an isothermal oven at 150 °C for 48 h, the white powder was collected via centrifugalization. The product was washed with DMF and ethanol, and then dried at 60 °C under vacuum.

### Synthesis of UiO-66-R

In all, 0.200 g of UiO-66-P was digested with 5 mL of 1 M NH_4_HCO_3_ aqueous solution. After that, 5 mL of 1 M CH_3_COOH aqueous solution was added into the digestion solution, followed by an evaporation process. Then, the white powdery residue and 3.154 g of benzoic acid (25.82 mmol) were dissolved in 6 mL of DMF in a vial. Subsequently, the solution was transferred into an autoclave and heated in an isothermal oven at 150 °C for 48 h. Then, the synthesized UiO-66-R was washed with DMF and ethanol three times, respectively, and finally dried under vacuum at 60 °C.

### Synthesis of PCN-224

In all, 0.06 g of ZrCl_4_ (1.73 mmol), 0.02 g of TCPP (0.86 mmol), and 0.80 g of benzoic acid (25.88 mmol) were dissolved in 4 mL of DMF in a pyrex vial and stirred until the solid was dissolved. Then, the solution was heated in an isothermal oven at 120 °C for 36 h. After centrifugalization, the product was washed with DMF and methanol three times, respectively, and finally dried under vacuum at 60 °C.

### Synthesis of PCN-224-R

In all, 0.020 g of PCN-224 was digested with 2 mL of 1 M NH_4_HCO_3_ aqueous solution. After that, 2 mL of 1 M CH_3_COOH aqueous solution was added into the digestion solution, followed by an evaporation process. Then, the dark-red powdery residue and 0.478 g of benzoic acid were dissolved in 2.5 mL of DMF in a vial. Subsequently, the solution was transferred into an autoclave and heated in an isothermal oven at 120 °C for 36 h. Then, the synthesized PCN-224-R was washed with DMF and ethanol three times, respectively, and finally dried under vacuum at 60 °C.

### Synthesis of Pd@PCN-224

In all, 0.025 g of PCN-224 was ultrasonically dispersed in 1 mL of ethylene glycol and then preheated at 120 °C for 10 min. After that, 47 μL of 0.6 M PVP and 23 μL of 6 mM PdCl_2_ aqueous solution was added to the ethylene glycol every 30 s with constant vigorous stirring (total volumes for PVP and PdCl_2_ solutions are 1500 and 750 μL, respectively). The resulting mixture was kept at 120 °C for an additional 10 min and then cooled down to room temperature. The obtained product was washed thoroughly several times with water and ethanol. The synthesized sample was further dried overnight under vacuum at 60 °C.

### Digestion experiment

Zr-MOFs were immersed in 1 M salt aqueous solution, after which ultrasonic treatment was performed for 20 min.

### TCPP recycle

Ten milligrams of PCN-224 was immersed in 3 mL of 1 M NH_4_HCO_3_ aqueous solution, followed by ultrasonic treatment. After complete digestion, 0.1 M HCl was added into the solution drop by drop until no more precipitation was generated. The products were collected by centrifugation and washed with water several times, and finally dried overnight at 80 °C.

### XRD measurement

XRD data were recorded on a Rigaku Miniflex600 diffractometer operated at 40 kV, 15 mA for Cu K_α_ (*λ* = 1.5406 Å) with a scan speed of 5°/min and a step size of 0.02° in 2*θ* at ambient temperature and pressure.

### N_2_ adsorption experiments

N_2_ adsorption experiments were measured on a micromeritics ASAP 2020 HD88 physisorption instrument. A liquid nitrogen bath (77 K) was used for isotherm measurements. High-purity grade N_2_ was used in the adsorption experiments. Before performing the analysis, the sample was outgassed at 150 °C under vacuum for 15 h. The multipoint BET analysis was performed by plotting 1/*v*(1 − *P*/*P*_0_) versus *P*/*P*_0_ (*P*_0_ = 1 bar) and *v* is the volume of nitrogen adsorbed per gram of MOF at STP. Pore size distributions for MOFs were analyzed by using DFT theory for N_2_ adsorption based on a carbon model.

### SEM imaging

SEM measurements were performed on a Zeiss Merlin Compact with an accelerating voltage of 5 kV. The samples were placed on clean aluminum foil and sputtered with platinum.

### Fourier transform-infrared spectra

All FTIR spectra were recorded on a Nicolet micro FTIR Specrometer iN10 and samples were tableted with KBr powder.

### Raman spectra

A Renishaw-RM1000 spectrometer equipped with a CCD detector was used for the Raman studies. A laser with a wavelength of 532 nm was applied for excitation. Raman spectra were gathered by using two scans of 10-s exposure each time.

### ^1^H NMR test

All of the ^1^H NMR data were collected on Bruker Avance III HD 400 MHz nuclear magnetic resonance spectrometer. For digestion, UiO-66 was immersed in D_2_O solution with 1 M NH_4_HCO_3_. Moreover, UiO-66 and BDC were immersed in D_2_O with NH_3_•H_2_O because of the poor solubility of BDC in water. For ligand recycle of PCN-224, the recycled TCPP was immersed in deuterated dimethylsulfoxide.

## Supplementary information

Supplementary Information

## Data Availability

The data supporting the findings of this study are available within the article and its Supplementary Information files, or from the corresponding authors on reasonable request.
