# Supplementary material for: Facile and reversible digestion and regeneration of zirconium-based metal-organic frameworks
Source: Commun Chem. 2020 Jan 9;3:5. doi: 10.1038/s42004-019-0248-7 (PMC9812265; doi:10.1038/s42004-019-0248-7)
Supplement: Supplementary file 1 — Supplementary Information [file 42004_2019_248_MOESM1_ESM.pdf]

Supplementary Information

Facile and reversible digestion and regeneration of zirconium-based metal-organic frameworks

Jun Chu[a], Fu-Sheng Ke[a], Yunxiao Wang[a], Xiangming Feng[b], Weihua Chen[b], Xinping Ai[a], Hanxi Yang[a], Yuliang Cao[a]*

[a] College of Chemistry and Molecular Science, Hubei Key Laboratory of Electrochemical Power Sources, Wuhan University, Wuhan 430072, China

[b] College of Chemistry and Molecular Engineering, Zhengzhou University, Zhengzhou 450001, China

*Correspondence: ylcao@whu.edu.cn

Supplementary Methods

1.1 Chemicals

Organic reagents: N, N-dimethylformamide (DMF), 1,4-benzenedicarboxylic acid (BDC), benzoic acid, ethanol, acetate acid, ammonium acetate, ammonium citrate tribasic and ethylene glycol were purchased from Sinopharm Chemical Reagent Co., LTD. 1,2,4-benzenetricarboxylic acid (BDC-COOH) and meso-tetra(4-carboxyphenyl)porphine (TCPP) were purchased from Energy Chemical (China). 1,4- naphthalenedicarboxylic acid was purchased from Tokyo Chemical Industry Co., LTD. 2-Bromoterephthalic acid (BDC-Br) was purchased from Adamas Reagent Co., LTD. Polyvinyl pyrrolidone (PVP, kw: 58000) was purchased from Shanghai Aladdin Bio-Chem Technology Co., LTD.

Inorganic reagents: zirconyl chloride octahydrate ($ZrOCl_2 \cdot 8H_2O$), ammonium bicarbonate (NH_4HCO_3), sodium bicarbonate ($NaHCO_3$), potassium bicarbonate ($KHCO_3$), ammonium carbonate ($(NH_4)_2CO_3$), lithium carbonate (Li_2CO_3), sodium carbonate (Na_2CO_3), potassium carbonate (K_2CO_3), hydrochloric acid (HCl), ammonium hydroxide ($NH_3 \cdot H_2O$), sodium hydroxide (NaOH), ammonium chloride (NH_4Cl), ammonium nitrate (NH_4NO_3), triammonium phosphate ($(NH_4)_3PO_4$), and trisodium phosphate (Na_3PO_4) were purchased from Sinopharm Chemical Reagent Co., LTD. Zirconium chloride ($ZrCl_4$) and palladium chloride ($PdCl_2$)were purchased from Shanghai Aladdin Bio-Chem Technology Co., LTD. Carbonic acid ammonium zirconium salt (AZC) was purchased from Energy Chemical (China).

All chemicals were used without further purifications.

1.2 Synthesis methods

Synthesis method of UiO-66-P, UiO-66-COOH, UiO-66-Br and UiO-66-NDC were modified from previous literatures.[1] Synthesis method of PCN-224 were modified from previous literatures.[2-3] Synthesis method of Pd@PCN-224 were modified from previous literatures.[3-4] All the details of synthesis are shown in main paper methods section.

The concentration of all salt aqueous solution in this paper are around 1 M. It is worth noting that the $LiHCO_3$ aqueous solution was produced by bubbling CO_2 into the

suspension liquid of Li_2CO_3 until the white precipitate disappeared and the solution became clear.

Supplementary Note 1

The possible mechanism of UiO-66 digesting in carbonate and citrate aqueous solution are as follow:

In carbonate solution:

$$Zr_6O_4(OH)_4(BDC)_6 + 18\ CO_3^{2-} + 10\ H_2O \rightarrow 3\ [Zr_2(OH)_2(CO_3)_4]^{2-} + 18\ OH^- + 6\ CO_2 + 6\ BDC^{2-} \quad (1)$$

$$OH^- + CO_2 \rightarrow HCO_3^- \quad (2)$$

$$CO_3^{2-} + CO_2 + H_2O \rightarrow 2\ HCO_3^- \quad (3)$$

In citrate solution:

$$Zr_6O_4(OH)_4(BDC)_6 + 8\ C_6H_8O_7^{3-} + 4\ H_2O \rightarrow 2\ (C_6H_5O_7)_4Zr_3 + 12\ OH^- + 6\ BDC^{2-} \quad (4)$$

Supplementary Note 2

The possible chemical reactions of UiO-66-R formation are as follow:

$$HCO_3^- + CH_3COOH \rightarrow CH_3COO^- + H_2O + CO_2 \quad (5)$$

$$[Zr_2(OH)_2(CO_3)_4]^{2-} + 10\ CH_3COOH \rightarrow 6\ H_2O + 2\ Zr^{4+} + 4\ CO_2 + 10\ CH_3COO^- \quad (6)$$

$$6\ Zr^{4+} + 24\ CH_3COO^- + 8\ H_2O + 6\ H_2BDC \rightarrow Zr_6O_4(OH)_4(BDC)_6 + 24\ CH_3COOH \quad (7)$$

Supplementary Figures

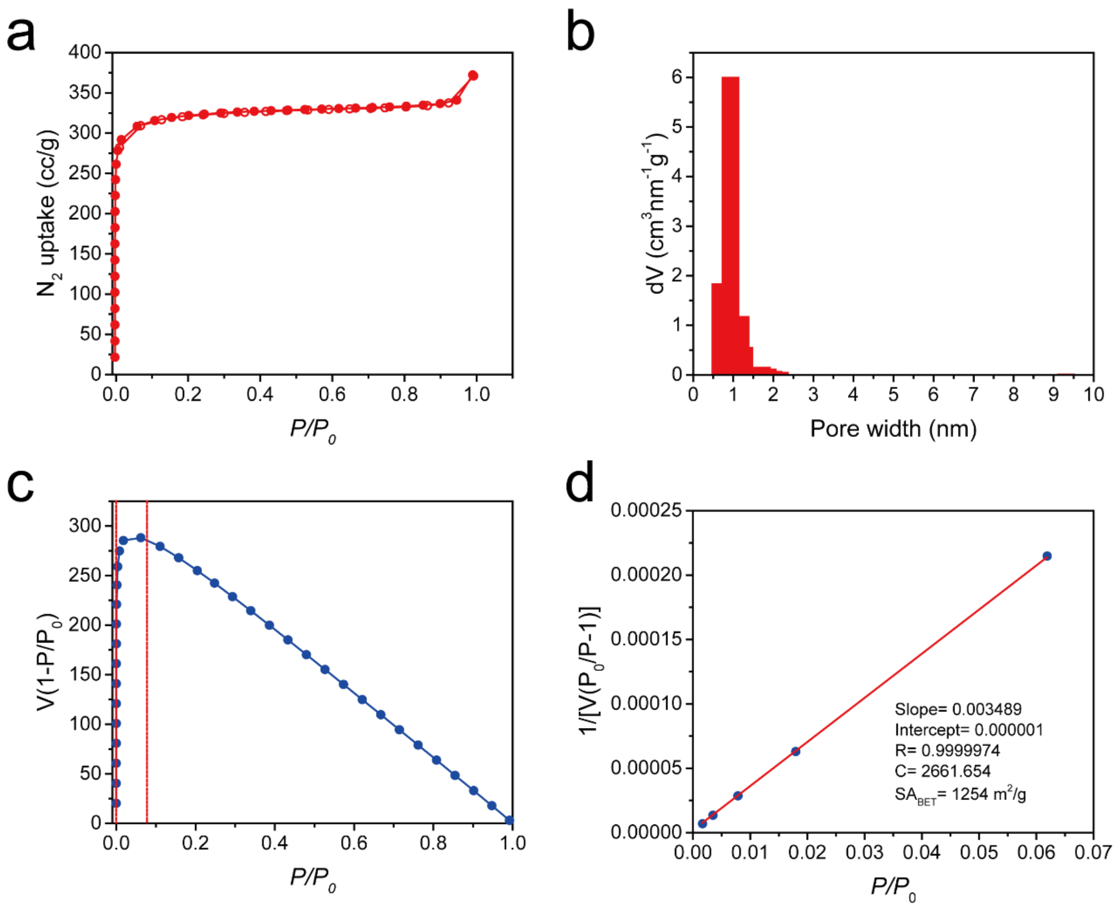

Supplementary Figure 1 A Isotherm of N_2 adsorption-desorption at 77 K and **b** pore size distribution calculated by DFT method for UiO-66-P. BET surface area calculation of UiO-66-P: **c** The points between red lines were selected based on the first consistency criterion, and **d** plot to select linear P/P_0 range.

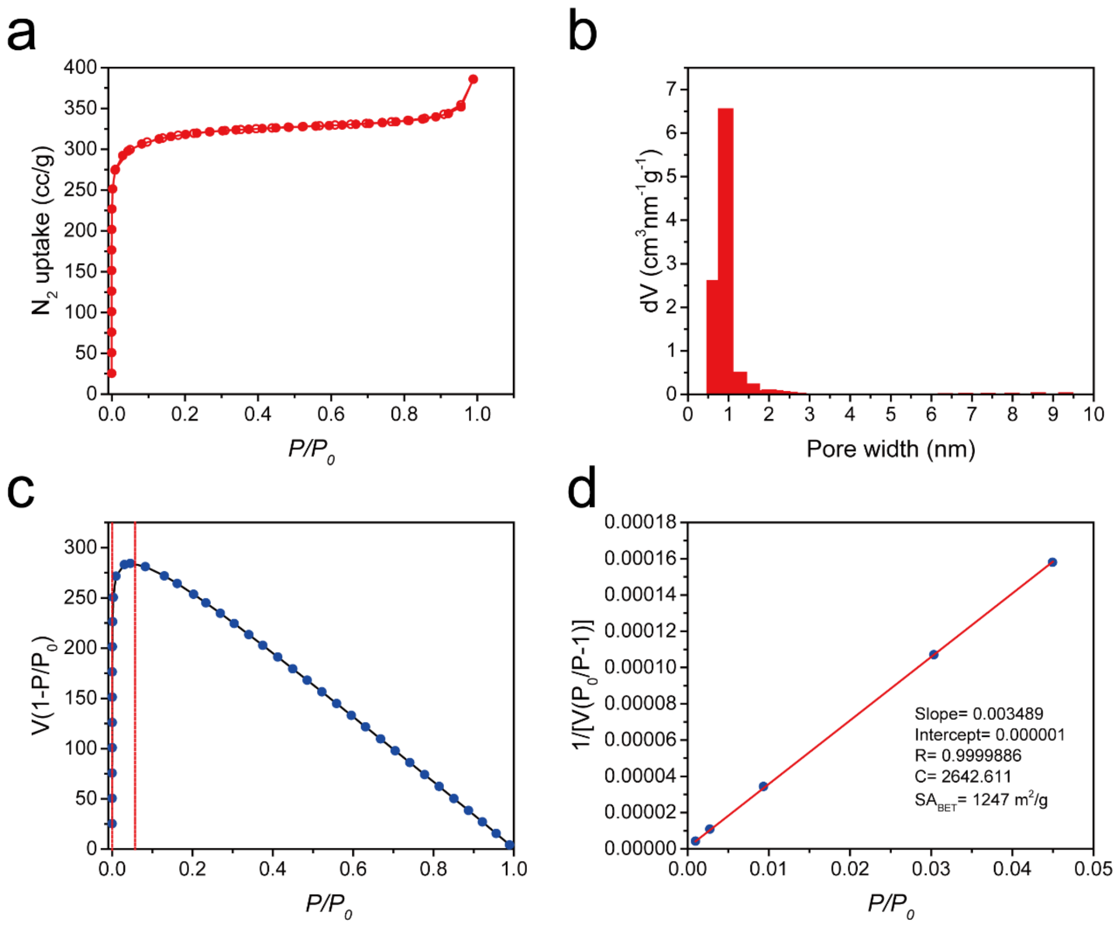

Supplementary Figure 2 A Isotherm of N_2 adsorption-desorption at 77 K and **b** pore size distribution calculated by DFT method for UiO-66-R. BET surface area calculation of UiO-66-R: **c** The points between red lines were selected based on the first consistency criterion, and **d** plot to select linear P/P_0 range.

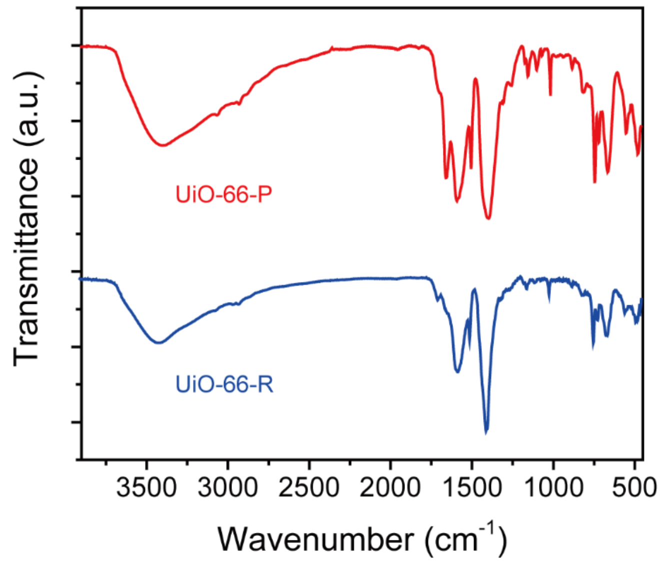

Supplementary Figure 3 FTIR spectra of as-synthesized UiO-66-P (red) and UiO-66-R (blue).

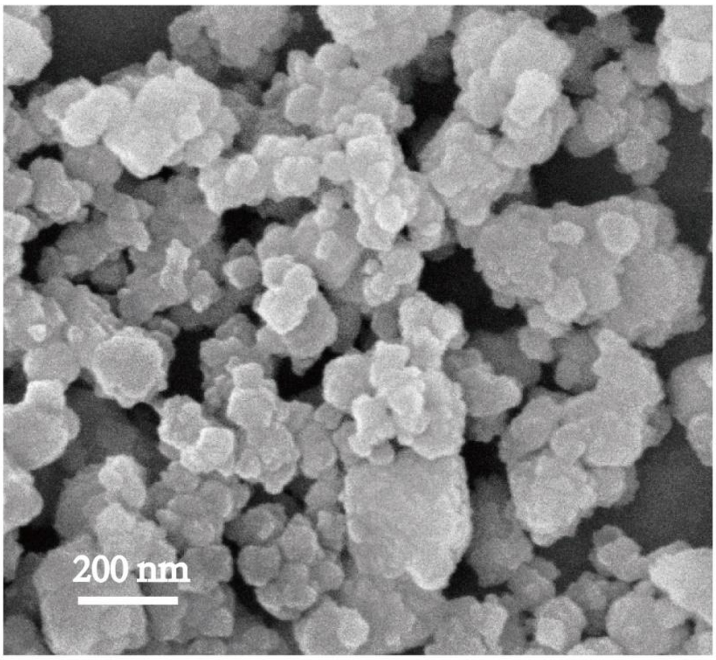

Supplementary Figure 4 SEM image of as-synthesized UiO-66 synthesized with trace amount of additional water.

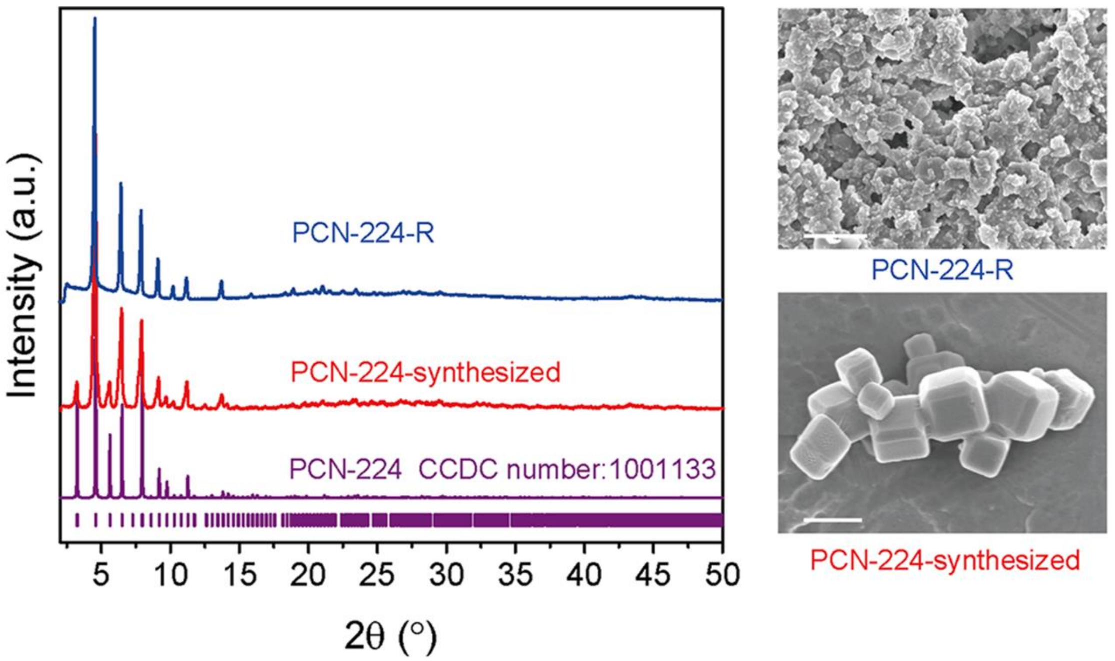

Supplementary Figure 5 XRD patterns of reported PCN-224 (purple, CCDC number: 1001133), PCN-224-synthesized (red), PCN-224-R (regenerated PCN-224, blue). And SEM images of PCN-224-synthesized (down) and PCN-224-R (up) (scale bar: 1 μm).

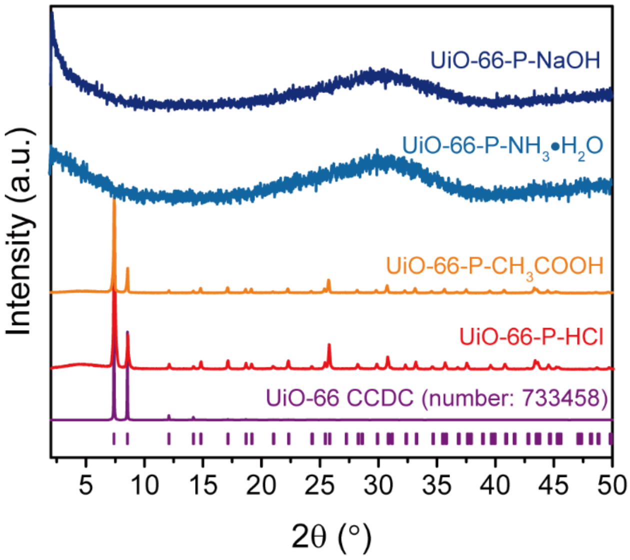

Supplementary Figure 6 XRD patterns of UiO-66-P immersed in: HCl (red), CH_3COOH (orange), NH_3•H_2O (light blue) and NaOH (dark blue). The concentration of these solutions is 1M.

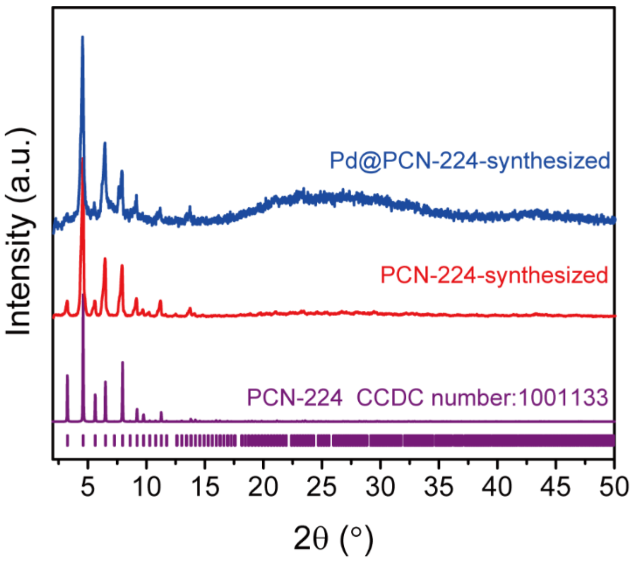

Supplementary Figure 7 XRD patterns of synthesized PCN-224 (red) and Pd@PCN-224 (blue).

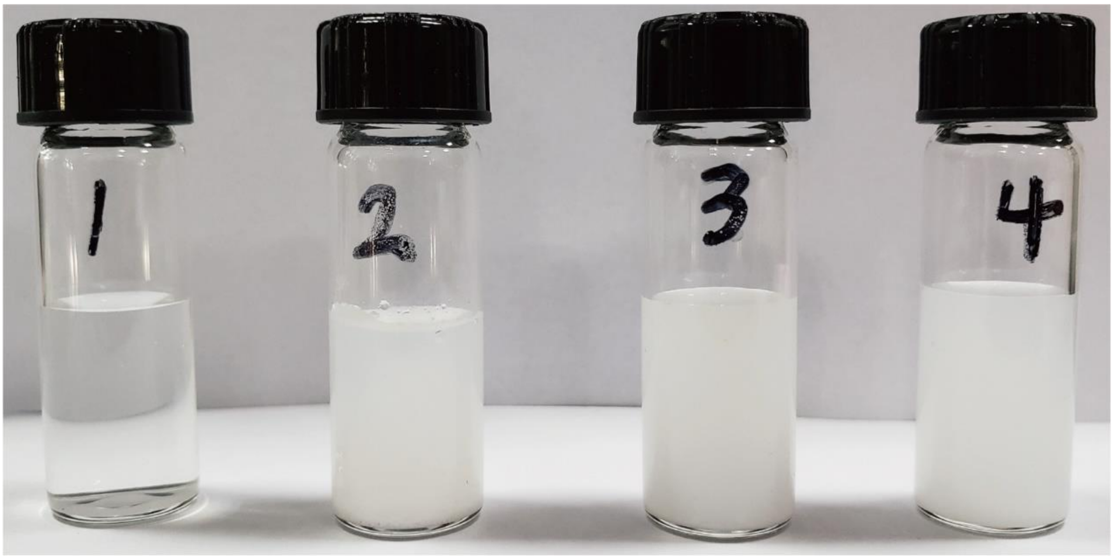

Supplementary Figure 8 UiO-66-P immersed in: 1) NH_4HCO_3, 2) CH_3COONH_4+NH_3•H_2O, 3) $NH_4H_2PO_4$+NH_3•H_2O and 4) Na_3PO_4•$12H_2O$+H_3PO_4 aqueous solution. All of them obtain the pH value around 7.6.

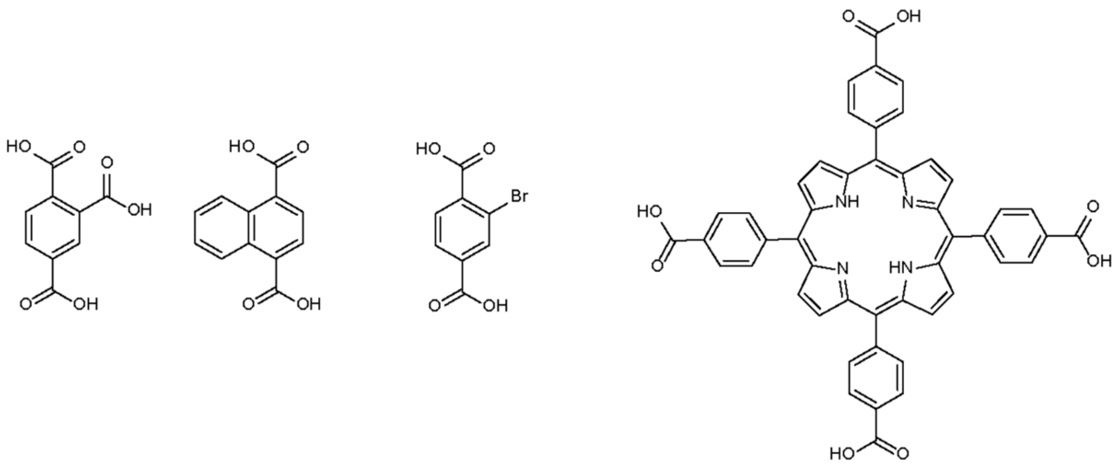

Supplementary Figure 9 Molecule structure illustrations of various ligands.

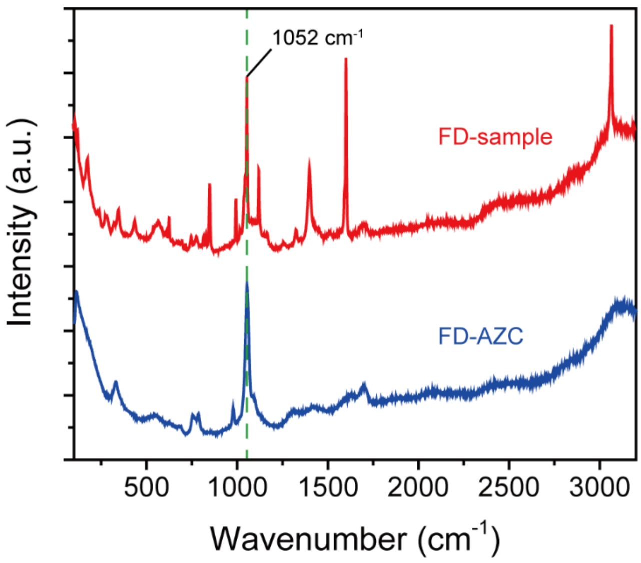

Supplementary Figure 10 Raman spectra of FD-sample (red) and FD-AZC (blue). The peak located in 1052 cm^{-1} indicated the existence of anion which consists of Zr^{4+} and CO_3^{2-}.

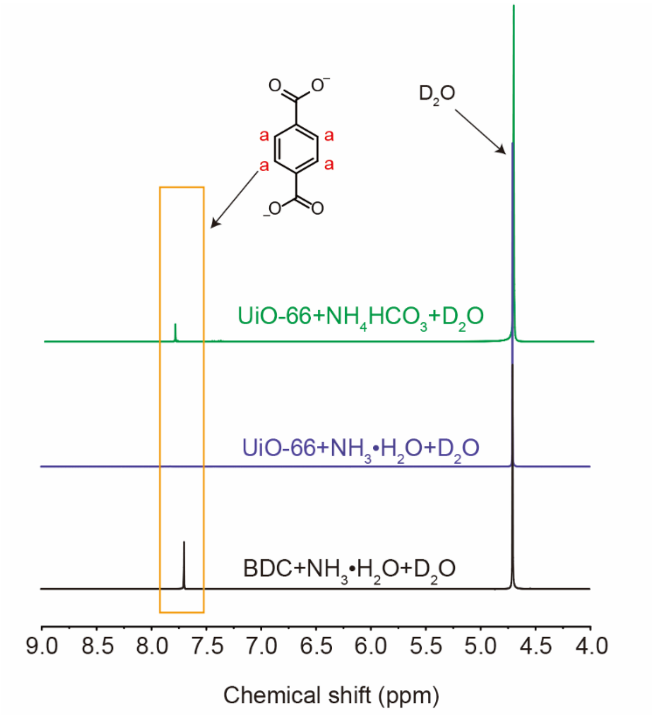

Supplementary Figure 11 ^{1}H NMR of UiO-66 in D_2O with and without NH_4HCO_3.

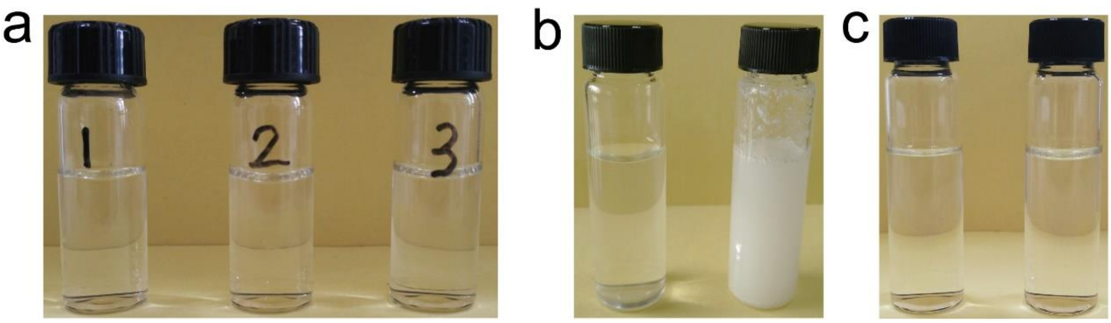

Supplementary Figure 12 UiO-66-P immersed in 1) $(NH_4)_2CO_3$, 2) Na_2CO_3 and 3) K_2CO_3 aqueous solution. **b** UiO-66-P immersed in NH_4HCO_3 (left) and ammonium citrate (right) after ultrasonic treatment for 20 min. **c** UiO-66-P immersed in NH_4HCO_3 (left) and ammonium citrate (right) after 3 days.

Supplementary References

1 Biswas S. et al. Enhanced selectivity of CO_2 over CH_4 in sulphonate-, carboxylate- and iodo-functionalized UiO-66 frameworks. *Dalton Trans.* **42,** 4730-4737 (2013).

2 Feng D. et al. Construction of ultrastable porphyrin Zr metal–organic frameworks through linker elimination. *J. Am. Chem. Soc.* **135,** 17105-17110 (2013).

3 Chen Y.-Z. et al. Singlet oxygen-engaged selective photo-oxidation over Pt nanocrystals/porphyrinic MOF: the roles of photothermal effect and Pt electronic state. *J. Am. Chem. Soc.* **139,** 2035-2044 (2017).

4 Yan T., Zhu L., Ju H. & Lei J. DNA-walker-induced allosteric switch for tandem signal amplification with palladium nanoparticles/metal–organic framework tags in electrochemical biosensing. *Anal. Chem.* **90,** 14493-14499 (2018).
